# Psychometric evaluation of the Chinese version of the BENEFITS-CCCSAT based on CTT and IRT: a cross-sectional design translation and validation study

**DOI:** 10.3389/fpubh.2025.1532709

**Published:** 2025-03-18

**Authors:** Chuang Li, Youbei Lin, Betul Tosun, Pin Wang, Hong Ye Guo, Cheng Rong Ling, Ran Qi, Qing Yue Luo, Yan Wang, Fang Huang, Jiaqi Wang, Shu Hui Ma, Dan Feng Xu, Shu Zhen Wu, Lan Zhang

**Affiliations:** ^1^First Affiliated Hospital of Jinzhou Medical University, Jinzhou, China; ^2^School of Nursing, Jinzhou Medical University, Jinzhou, China; ^3^Faculty of Nursing, Hacettepe University, Ankara, Türkiye; ^4^The First People’s Hospital of Shenyang, Shenyang, China; ^5^Department of Nursing, The Second People’s Hospital of Yibin, Yibin, China; ^6^School of Nursing, Jinzhou Normal College, Jinzhou, China; ^7^School of Nursing, Liaodong University, Dandong, China; ^8^School of Nursing Taizhou University, Taizhou, China

**Keywords:** cross-cultural nursing, nursing students and education, psychometrics (MeSH), classical test theory, item response theory

## Abstract

**Background:**

The importance of culturally competent care in multicultural environments is increasingly recognized; however, effective tools to assess nursing students’ cross-cultural competence remain limited. This study aimed to validate the BENEFITS-CCCSAT for Chinese nursing students.

**Methods:**

The original BENEFITS-CCCSAT was translated, back-translated, culturally adapted, and pre-tested using the Brislin model to form a Chinese version. A combined approach of classical test theory (CTT) and item response theory (IRT) was then used for multidimensional validation.

**Results:**

The CTT analysis showed that the C-BENEFITS-CCCSAT had a Cronbach’s *α* coefficient of 0.80, dimension reliability values ranging from 0.700 to 0.905, a test–retest reliability value of 0.881, and a scale-level content validity index (S-CVI) value of 0.928. The criterion-related validity value was 0.619. The confirmatory factor analysis (CFA) indicated a good model fit (CMIN/DF = 1.071, RMSEA = 0.08), with factor loadings ≥0.50. The Rasch analysis showed an item reliability value of 1, person reliability values ranging from 0.76 to 0.89, item separation index values ranging from 17.37 to 60.34, and person separation index values ranging from 1.76 to 2.89. The information-weighted fit statistic mean square (infit MNSQ) and outlier-sensitive fit statistic mean square (outfit MNSQ) values for all items ranged from 0.86 to 1.27. Overall, the scale demonstrated good reliability and validity for the Chinese nursing students.

**Conclusion:**

The 25-item C-BENEFITS-CCCSAT demonstrates good reliability and validity and can be applied in educational settings to assess students’ ability to provide culturally competent care. Future studies should test the scale in culturally diverse populations to further determine its applicability and generalizability.

## 1 Introduction

The world is currently experiencing an unprecedented rate of population mobility, driven by rapid advancements in the information age and the accelerated movement of people worldwide (1). In this context, the “Belt and Road” initiative has been introduced and widely promoted, further accelerating the internationalization of Chinese society. As a result, an increasing number of expatriates are coming to China for work, academic exchanges, and study (2). According to China’s seventh population census, approximately 376 million people now constitute the floating population (3). Therefore, significant challenges and unique opportunities for transcultural nursing are posed by this increase in cultural diversity. Introduced by Madeleine Leininger, a renowned American nursing theorist, in the 1960s, the concept of transcultural nursing emphasizes its core principle of cultural care. Advocated by Leininger, this concept stresses that nurses should provide care that is safe, effective, and aligned with the values, beliefs, customs, and lifestyles inherent to the cultural backgrounds of their patients—elements shared, preserved, and transmitted across generations within each cultural group (4). Leininger’s Sunrise Model conceptualizes transcultural nursing through four interconnected levels, each elucidating a distinct theoretical component and its relationships with the others. Encompassing worldviews, cultural norms, and social structures, the highest level addresses individuals’ diverse perspectives and unique ways of life. Referred to as the service-object level, the second level highlights how individuals from specific cultures express their thoughts, emotions, and practices related to health and care. Focused on the healthcare system, the third level includes unique folk care practices within cultural groups. Finally, representing a targeted approach to nursing care, the fourth level—the decision-making and action level—emphasizes culturally congruent interventions (5).

As a critical workforce in the healthcare industry, nurses must possess cultural competence to address the complexities arising from diverse cultures, traditions, dietary practices, religious beliefs, and thought processes encountered in daily nursing practice (6). Underscoring this need, the urgent call for transcultural nursing education arises, aiming to equip nursing students with the necessary knowledge, skills, and assessment tools to deliver culturally competent care (7). However, a systematic review examining the relationship between educational strategies promoting cultural competence and patient treatment outcomes revealed a lack of standardized approaches. The absence of systematic educational strategies and assessment methods has led to inconclusive effects on improving cultural competence among healthcare professionals (8). Many existing assessment tools are based on specific theoretical models. For instance, Choi and Kim (9) utilized the “Nursing Students’ Cultural Competence Scale” in their study, while Ge Yunyun developed the Cross-Cultural Sensitivity Scale for nursing students in 2006 (10). These tools highlight efforts to measure and enhance cultural competence but also reflect the need for more consistent and validated methodologies.

In the context of a multicultural environment, effective nursing education aimed at enhancing transcultural nursing skills requires robust evaluation tools. The better and effective nursing education for improving transcultural nursing skills cultural competence and cultural sensitivity assessment tool (BENEFITS-CCCSAT) integrates the strengths and features of existing instruments, providing a comprehensive framework for assessing the outcomes of cross-cultural nursing education, as well as cultural competence and sensitivity (7). In contrast, transcultural sensitivity measurement tools developed independently by Chinese scholars remain limited, both in their scope of application and in the scientific validation of their effectiveness (11). To address this gap, the present study aimed to adapt the BENEFITS-CCCSAT for the Chinese context, evaluate its reliability and validity, and explore its applicability and potential value in assessing and fostering cultural competence among nursing students in China.

## 2 Method

### 2.1 Study design

This research was a cross-sectional, multicenter study.

### 2.2 Data collection

The conclusions of this study were drawn from the data collected between April and August 2024. A total of 1,074 nursing students, recruited through convenience sampling from eight medical universities in Northeast, Southwest, and North China, participated. The survey was distributed via the China Questionnaires Star platform^1^ and administered under standardized instructions. The students were informed about the study’s purpose, the scale’s instructions, and the estimated time for completion, and those who provided consent were included. To ensure data integrity, the e-questionnaire could only be submitted after all items were fully completed, and only one submission per participant was allowed. The sample size was estimated using the Kendall method, based on the recommendation of 5–10 times the number of items in the questionnaire, while accounting for a potential sample loss rate of 10–20% (12). With 26 items in the questionnaire, the required sample size was calculated to range from 143 to 312 participants. To meet the sample size threshold for confirmatory factor analysis (CFA) (minimum of 200 cases) and ensure the study’s generalizability (13), approximately 1,200 questionnaires were distributed. After excluding the responses with completion times under 3 min and those with obviously patterned answers, 1,074 valid questionnaires were retained, resulting in a recovery rate of 89.5%.

### 2.3 Participants

The inclusion criteria were as follows: (1) Full-time college degree or above; (2) voluntary participation in the research; (3) previous clinical apprenticeship or internship experience, and (4) knowledge about basic nursing skills and intervention measures. The exclusion criteria were as follows: (1) Students with mental illness, such as emotional disorder and depression, as it is considered that students with mental illness may experience serious emotional or cognitive difficulties, which make it difficult to answer questions.

### 2.4 Tools

#### 2.4.1 The researcher created a general information questionnaire

The data collected included information on educational level, grade, sex, age, ethnicity, home residence, and whether participants had attended any courses or training related to cross-cultural care.

#### 2.4.2 Chinese version of the BENEFITS–CCCSAT (C-BENEFITS-CCCSAT)

It consists of five dimensions with 25 items, as follows: respect for cultural diversity (6 items), challenges and barriers providing culturally competent care (3 items), achieving cultural competence (3 items), culturally sensitive communication (5 items), and perceived meaning of cultural care (8 items). Seven items (items 7, 8, 9, 14, 15, 17, and 18) are reverse scored. A seven-point Likert scale is used, with one representing “strongly disagree” and seven representing “strongly agree.” The overall score ranges between 25 and 175, with higher scores indicating greater cultural competence, cultural sensitivity, and intercultural nursing skills among nursing students.

#### 2.4.3 Chinese version of the short form of the cultural competence scale for nurses (C-SFCCSN)

The instrument, developed by Chae et al. (14) and translated by Yajin Zhu (15), was designed to assess the cultural competence of clinical nurses. It consists of four dimensions: cultural awareness (6 items), cultural knowledge (7 items), cultural sensitivity (13 items), and cultural skills (7 items), for a total of 33 items. Each item is scored on a 7-point Likert scale ranging from 1 (strongly disagree) to 7 (strongly agree). The total score can range from 33 to 231, with higher scores indicating greater cultural competence in clinical nurses.

### 2.5 Translation and cultural adaptation of the BENEFITS-CCCSAT

The BENEFITS-CCCSAT was developed by Ayla (7). It consists of five dimensions with 26 items, as follows: respect for cultural diversity (1–6 items), challenges and barriers providing culturally competent care (7–10 items), achieving cultural competence (11–13 items), culturally sensitive communication (14–18 items), and perceived meaning of cultural care (19–26 items). A seven-points Likert scale is used, with one representing “strongly disagree” and seven representing “strongly agree.” The overall score is between 26 and 182, with higher scores indicating greater cultural competence, cultural sensitivity, and intercultural nursing skills among nursing students. In addition, eight items (items 7, 8, 9, 10, 14, 15, 17, and 18) are reverse scored. The BENEFITS-CCCSAT has good construct validity and reliability, with an internal consistency Cronbach’s *α* coefficient of 0.828.

To obtain permission to use the scale, the research team contacted the original developers via email. The translation process closely followed the Brislin translation model (16).

#### 2.5.1 Step 1

The original English version of the scale was translated into two Chinese versions (B1 and B2) by two native Chinese speakers who were nursing master’s degree candidates. The research team then reviewed and discussed the two Chinese versions, resolving any discrepancies and combining them into a preliminary Chinese version, B3.

#### 2.5.2 Step 2

Two native Chinese educators, unfamiliar with the questionnaire, independently translated the Chinese version B3 back into English (producing versions TB1 and TB2). These versions were then combined to form the English version TB3. The original authors were asked to review TB3 and provide feedback. The research team further deliberated and made revisions before finalizing the Chinese version of the scale, F1.

#### 2.5.3 Step 3

To culturally adapt the Chinese version F1, the Delphi method was employed. Six experts were recruited: two clinical nursing experts and four nursing education specialists, all with associate senior titles and at least a master’s degree (two with master’s degrees and four with doctoral degrees). The experts, with an average age of 35.43 ± 5.53 years and an average of 13.14 ± 4.02 years of relevant experience, assessed the cultural relevance, contextual appropriateness, and linguistic expression of the items in the Chinese version F1, comparing them with the original scale. Based on the experts’ feedback, the research team finalized the questionnaire through necessary revisions.

#### 2.5.4 Step 4

A total of 30 randomly selected nursing students were given a questionnaire for the presurvey, and all the students responded that all the items on the scale were comprehensible.

### 2.6 Ethical statement

The study was approved by the Ethics Review Committee of Jinzhou Medical University (JZMULL2025043) and adhered to their ethical guidelines. Informed consent was obtained from all participating students to ensure their confidentiality and anonymity. The study was conducted in accordance with the ethical principles outlined in the Declaration of Helsinki.

### 2.7 Statistical analysis

Statistical analysis was performed using SPSS 27.0, AMOS 26.0, and Winsteps 3.72.3. Prior to the analysis, the data were cleaned to address invalid and missing values. Descriptive statistics, including mean and standard deviation (mean ± SD), were used to summarize the quantitative data following a normal distribution, while frequency and percentage (%) were used to describe the qualitative data.

#### 2.7.1 Analysis of the CTT model

This study involved the analysis of classical test theory (CTT) data, including assessments of the reliability and validity of the scales. Reliability was evaluated through internal consistency, split-half reliability, and test–retest reliability (17). Regarding validity, content validity was established using the Delphi method (18), while structural validity was assessed through confirmatory factor analysis (CFA), which evaluated convergent and discriminant validity. The validity of the calibration correlations was determined using the Pearson correlation test between the C-SFCCSN and the C-BENEFITS-CCCSAT (19).

#### 2.7.2 Analysis of the IRT model

The validity of the Rasch analysis requires confirmation of unidimensionality, which was evaluated through principal component analysis (PCA) of residuals (20). Reliability was assessed using person and item separation indices to determine the scale’s discriminative capacity across dimensions, alongside person and item reliability metrics. Item fit was examined using the following indices (21): ① Information-weighted fit statistic mean square (infit MNSQ), ② outlier-sensitive fit statistic mean square (outfit MNSQ), and ③ point-measure correlation (PT-Measure Corr). The residual patterns were further analyzed via PCA. Item-person fit was visualized using Wright maps, and the appropriateness of the response category thresholds was verified through item characteristic curves. Finally, differential item functioning (DIF) analysis was conducted to identify potential group-based measurement bias.

## 3 Results

### 3.1 Demographic characteristics

A total of 1,074 students participated in the validation study, with a mean age of 20.48 years (range:17–34; SD = 1.986). The sample consisted predominantly of female (68.5%) and undergraduate students (50.0%). Second-year students represented the largest group (55.8%). Detailed demographic information is provided in Table 1.

**Table 1:** Distribution of the participant attributes and demographic data.

Variables		Frequency (*n* = 1,074)	Percentage (%)
Sex	Male	338	31.5
	Female	736	68.5
Educational level	College students	328	30.5
	Undergraduate students	537	50.0
	Graduate students	209	19.5
Grade	First	134	12.5
	Second	599	55.8
	Third	307	28.6
	Fourth	34	3.2
Ethnicity	Han	712	66.3
	National minority	362	33.7
Home residence	Countryside	576	53.6
	Municipalities	498	46.4
WPHACOTCCC	Yes	493	45.9
	No	581	54.1

WPHACOTCCC, whether participants had attended any courses or training related to cross-cultural care.

### 3.2 CTT model results

#### 3.2.1 Item analysis

The recovered scales were sorted by total score, from high to low, with the top 27% defined as the high group and the bottom 27% as the low group. A normality test was then conducted, with skewness and kurtosis values between −2 and + 2 indicating that the data followed a normal distribution (22). A test of 579 cases confirmed that the data adhered to a normal distribution. Item analysis was performed using the two independent samples *t*-test, which revealed critical ratios ranging from 5.180 to 19.579 (all >3) and a *p*-value of <0.001 (23), indicating statistical significance. As a result, the items were considered to be well differentiated (Table 2).

**Table 2:** The C-BENEFITS-CCCSAT values for skewness, kurtosis, and the critical ratio.

Items	Mean (SD)	Skewness/Kurtosis	Critical ratio	*95% CI*	
Rfcd1	3.62(1.72)	0.144/−0.923	14.531	1.540	2.022
Rfcd2	4.56(1.70)	−0.354/−0.801	13.188	1.392	1.879
Rfcd3	3.44(1.72)	0.331/−0.836	12.951	1.387	1.883
Rfcd4	4.50(1.64)	−0.242/−0.730	12.959	1.320	1.792
Rfcd5	4.15(1.70)	−0.089/−0.886	12.442	1.318	1.812
Rfcd6	4.93(1.60)	−0.491/−0.648	12.664	1.266	1.731
CB7	1.68(0.89)	1.414//1.809	5.180	0.235	0.523
CB8	4.54(1.65)	−0.253/−0.782	5.755	0.506	1.031
CB9	5.52(1.38)	−0.893/0.269	5.210	0.365	0.806
Acc11	3.27(1.66)	0.385/−0.755	8.579	0.861	1.372
Acc12	4.09(1.70)	−0.005/−0.927	6.662	0.641	1.177
Acc13	2.71(1.48)	0.716/−0.197	6.378	0.525	0.993
Csc14	3.81(1.69)	0.168/−0.939	11.398	1.200	1.700
Csc15	4.01(1.74)	−0.022/−0.936	10.725	1.163	1.684
Csc16	4.65(1.62)	−0.306/−0.716	11.271	1.140	1.621
Csc17	2.83(1.55)	0.632/−0.415	9.386	0.893	1.365
Csc18	5.02(1.54)	−0.571/−0.391	10.236	0.980	1.445
Pmoc19	3.92(1.76)	0.013/−0.936	19.355	1.986	2.435
Pmoc20	3.64(1.79)	0.189/−1.044	17.926	1.912	2.383
Pmoc21	5.14(1.61)	−0.584/−0.684	16.555	1.611	2.045
Pmoc22	3.70(1.76)	0.252/−0.936	18.500	1.926	2.383
Pmoc23	3.30 (1.77)	0.416/−0.893	18.669	1.942	2.399
Pmoc24	4.44(1.71)	−0.202/−0.928	17.417	1.785	2.239
Pmoc25	3.74(1.81)	0.076/−1.024	19.579	2.063	2.523
Pmoc26	5.01(1.67)	−0.568/−0.614	17.144	1.725	2.171

#### 3.2.2 Reliability

The Cronbach’s *α* coefficient for the C-BENEFITS-CCCSAT was 0.80, and the reliability of the individual dimensions were 0.880, 0.700, 0.759, 0.840, and 0.905, respectively. The split-half reliability was 0.837. Two weeks later, 40 students were randomly selected for a test–retest reliability assessment, and the test-retest reliability of the C-BENEFITS-CCCSAT was 0.881 (Table 3). All values were greater than the reference value of 0.7 (24), indicating good internal consistency and measurement invariance.

**Table 3:** Reliability, convergent, and discriminant validity of the C-BENEFITS-CCCSA.

Dimensions	Cronbach’s alpha coefficient	Split-half reliability	Test–retest reliability	Convergent Validity		Fornell–Larcker test				
				CR	AVE	F1	F2	F3	F4	F5
F1	0.880	0.837	0.881	0.880	0.550	**0.741**	–	–	–	–
F2	0.700	–	–	0.700	0.420	0.004	**0.648**	–	–	–
F3	0.759	–	–	0.761	0.515	−0.018	−0.023	**0.717**	–	–
F4	0.830	–	–	0.841	0.514	0.003	0.000	0.039	**0.716**	–
F5	0.905	–	–	0.870	0.500	−0.014	−0.015	0.067	0.000	**0.707**

The square root of the AVE is the value on the diagonal of the Fornell–Larcker test; F1: Respect for cultural diversity; F2: Challenges and barriers providing culturally competent care; F3: Achieving cultural competence; F4: Culturally sensitive communication; F5: Perceived meaning of cultural care. Bold values represents the square root of the AVE.

#### 3.2.3 Content validity

Six experts (two clinical experts and four nursing education experts) were invited to evaluate the cultural appropriateness and relevance of each item in the C-BENEFITS-CCCSAT. The expert panel consisted of four Ph.D. holders and two M.D. holders, all holding the title of Associate Professor or higher, with professional experience ranging from 10 to 21 years. A 4-point Likert scale was employed, where “Not Relevant” was scored as 1, “Weakly Relevant” as 2, “More Relevant” as 3, and “Very Relevant” as 4. The results indicated that the content validity index of the expert evaluation scale (S-CVI) was 0.928(>0.9) and the content validity index for each item (I-CVI) ranged from 0.83 to 1.00(>0.78) (25).

#### 3.2.4 Structural validity

**Figure 1:**
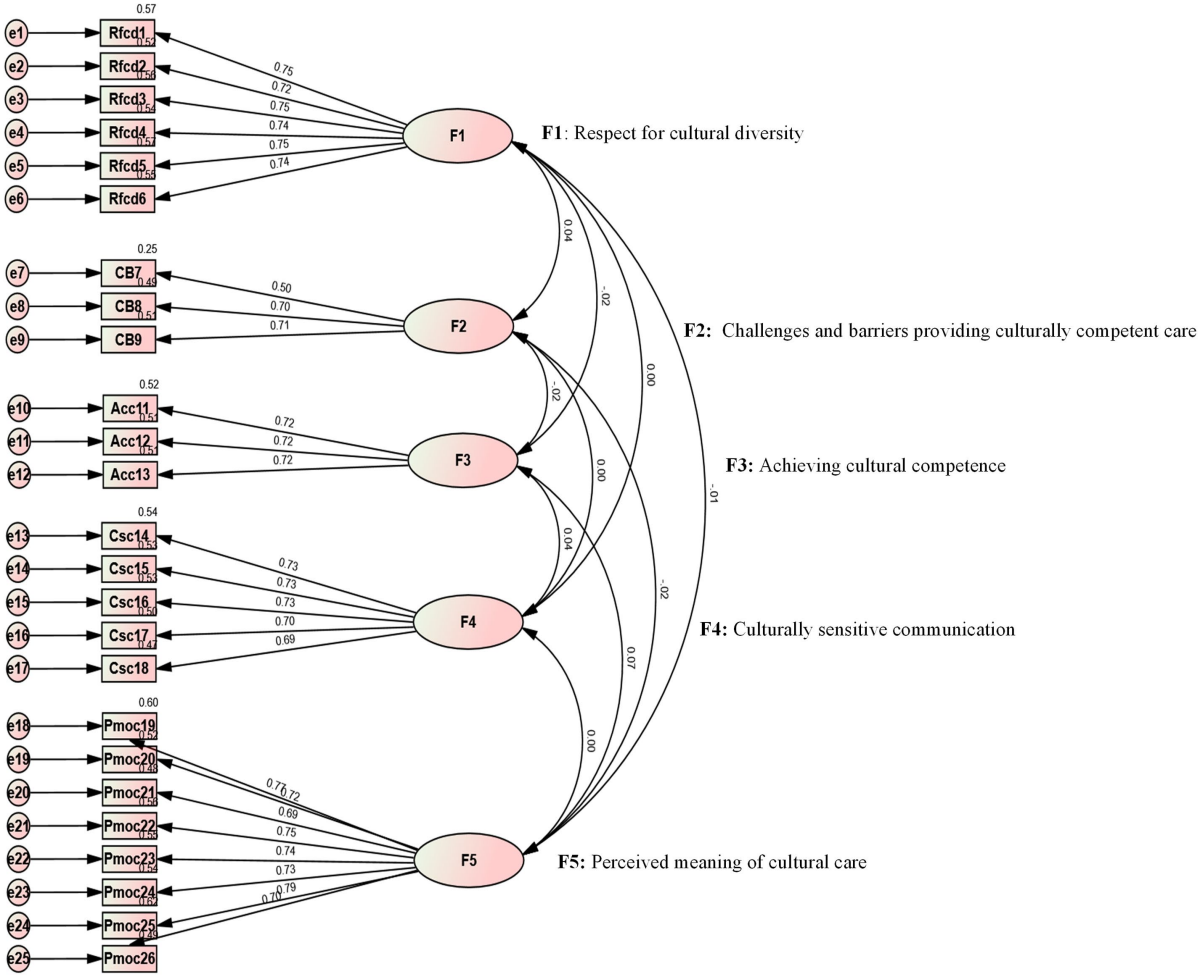
Five-factor standardized factor loadings plot of the C-BENEFITS-CCCSAT (*n* = 1,074).

The prerequisite for conducting factor analysis was calculating the KMO value and performing Bartlett’s test of sphericity. The results showed that the KMO value for the C-BENEFITS-CCCSAT was 0.884, which was greater than 0.7 (26). The Bartlett’s test of sphericity yielded a chi-square value of χ^2^ = 10,503.709 with *df* = 300, indicating a statistically significant difference (*p* < 0.001) and confirming the suitability of the data for factor analysis. Confirmatory factor analysis (CFA) was performed to assess the item-factor structure of the scale, and the model was estimated using the robust maximum likelihood method. The model showed a chi-square/degrees of freedom (CMIN/DF) ratio of 1.071 (less than 3), a root mean square error of approximation (RMSEA) value of 0.08 (≤0.08), and the following fit indices: the Comparative Fit Index (CFI) = 0.998, Normed Fit Index (NFI) = 0.973, Incremental Fit Index (IFI) = 0.998, and Tucker-Lewis Index (TLI) = 0.998, all greater than 0.9 (27). These results suggested that the model fit was good and that the factor loadings for all items were ≥ 0.50 (28) (Figure 1).

#### 3.2.5 Convergent and discriminant validity

Based on the standardized factor loadings from the CFA, the composite reliability (CR) values ranged from 0.700 to 0.880, all ≥0.7 (29). The average variance extracted (AVE) values ranged from 0.420 to 0.55, all >0.4 (30). Discriminant validity was tested using the Fornell–Larcker criterion, which revealed that the square root of the AVE value for each latent variable was greater than the correlation coefficients between that latent variable and the other latent variables (31). This indicated that the C-BENEFITS-CCCSAT demonstrated good convergent and discriminant validity (Table 3).

#### 3.2.6 Calibration correlation validity

Calibration correlation validity assesses the degree of correlation between a new instrument and an existing, authoritative, validated scale (32). A higher correlation coefficient indicates better validity for the new instrument. In this study, the C-SFCCSN was used as the reference standard for intercultural competence. The results showed that the calibration correlation validity of the C-BENEFITS-CCCSAT was 0.619 (33), which was greater than 0.5 but less than 0.7, indicating that the calibration correlation validity of this questionnaire for use with a population of Chinese nursing students is of moderate relevance.

### 3.3 IRT model results

#### 3.3.1 Unidimensionality test

The PCA of the residuals indicated a first principal component standardized residual value of 5.6 (>3.0) (34), suggesting multidimensionality, which violates the assumptions of the Rasch model. This could lead to a poorer fit and inaccurate estimation of the scale. However, Van der Linden argued that despite the potential multidimensionality of the overall scale (35), the Rasch model can still be applied, particularly when the dimensions are clearly defined. Specifically, the Rasch model not only evaluates the fit of each item but also analyzes the independence and validity of each dimension when the model is appropriately configured. Therefore, even if the scale is multidimensional, analyzing each dimension separately ensures that it accurately measures the underlying concept while avoiding inter-dimensional interference, thereby maintaining the validity of the measurement. In addition, related studies (36, 37) have indicated that dimensional analyses using the Rasch model can effectively capture independent information across multiple dimensions of the scale, successfully identifying underlying constructs. Drawing from these perspectives, we argue that when a scale exhibits multidimensionality, it suggests that the construct being measured encompasses multiple independent yet related sub-concepts. Each dimension may reflect a distinct aspect or domain, with items within each dimension measuring specific characteristics of that domain. Although the overall scale does not meet the unidimensionality assumption, it remains suitable for measurement as long as each individual dimension satisfies Rasch’s unidimensionality requirement. Therefore, based on the five dimensions classified by the original authors, a unidimensionality test was conducted to isolate the contribution of each dimension and ensure that each dimension’s effect was accurately estimated. The results showed that the standardized residual value of the first component for each dimension was less than 3.0 (Table 4), indicating that each dimension met the unidimensionality assumption and that there was no cross-dimensional overlap. This suggests that the scale remains a valuable tool for assessing transcultural nursing competencies.

**Table 4:** Overall scale and Rasch residual analyses of each dimension.

Dimension	Eigenvalue of first contrast residual	Explanatory variance (%)
Overall Scale	5.6	35.0
Respect for cultural diversity,	1.3	66.6
Challenges and barriers providing culturally competent care	1.7	87.7
Achieving cultural competence	1.6	71.7
Culturally sensitive communication	1.3	69.5
Perceived meaning of cultural care	1.3	66.8

#### 3.3.2 Reliability and separation indices

After conducting the Rasch analysis, the item reliability for each dimension of the C-BENEFITS-CCCSAT was 1.00, and the person reliability ranged from 0.76 to 0.86, both surpassing the critical threshold of 0.7 (38). The item separation indices ranged from 17.37 to 60.34, while the person separation indices ranged from 1.76 to 2.89, both exceeding the minimum acceptable value of 1.5 (39). These results indicate that both the sample and the items were well represented (Table 5).

**Table 5:** Analysis of the fit of the C-BENEFITS-CCCSAT and separation indices and reliability values for each dimension.

Items	INFIT MNSQ	OUTFIT MNSQ	PT-Measure Corr	Person		Item	
				Reliability	Separation	Reliability	Separation
Rfcd1	0.97	0.97	0.79	0.86	2.58	1.00	17.37
Rfcd2	1.05	1.03	0.77				
Rfcd3	0.96	0.95	0.79				
Rfcd4	0.99	1.01	0.78				
Rfcd5	0.97	0.97	0.79				
Rfcd6	0.96	0.97	0.77				
CB7	0.98	1.27	0.65	0.76	1.76	1.00	60.34
CB8	0.94	0.93	0.84				
CB9	0.91	0.97	0.81				
Acc11	1.00	0.99	0.81	0.76	1.80	1.00	21.08
Acc12	0.95	0.94	0.83				
Acc13	0.99	0.99	0.79				
Csc14	0.97	0.97	0.78	0.83	2.24	1.00	24.95
Csc15	1.01	1.01	0.78				
Csc16	0.97	0.96	0.78				
Csc17	0.96	0.97	0.76				
Csc18	1.00	1.05	0.75				
Pmoc19	0.93	0.94	0.79	0.89	2.89	1.00	20.95
Pmoc20	1.04	1.03	0.76				
Pmoc21	1.02	1.02	0.72				
Pmoc22	0.97	0.97	0.77				
Pmoc23	1.00	0.99	0.77				
Pmoc24	1.00	0.99	0.76				
Pmoc25	0.87	0.86	0.81				
Pmoc26	1.08	1.13	0.73				

#### 3.3.3 Fit of the items

Some researchers have proposed that the ideal criteria for the fit of item analysis include infit MNSQ and outfit MNSQ values between 0.6 and 1.4 (34), which indicate a good fit to the model in item analysis. In this study, the infit MNSQ values for each item of the C-BENEFITS-CCCSAT ranged from 0.87 to 1.08, and the outfit MNSQ values ranged from 0.86 to 1.27. The correlation coefficient measures how closely the items align with the measurement target, with an acceptable minimum value of 0.5 (32). In this study, the PT-Measure Corr values ranged from 0.65 to 0.84, exceeding the minimum reference value, suggesting that the scale items were closely aligned with the measurement target and that the study data fit well with the model (Table 5).

#### 3.3.4 Item-person matching

**Figure 2:**
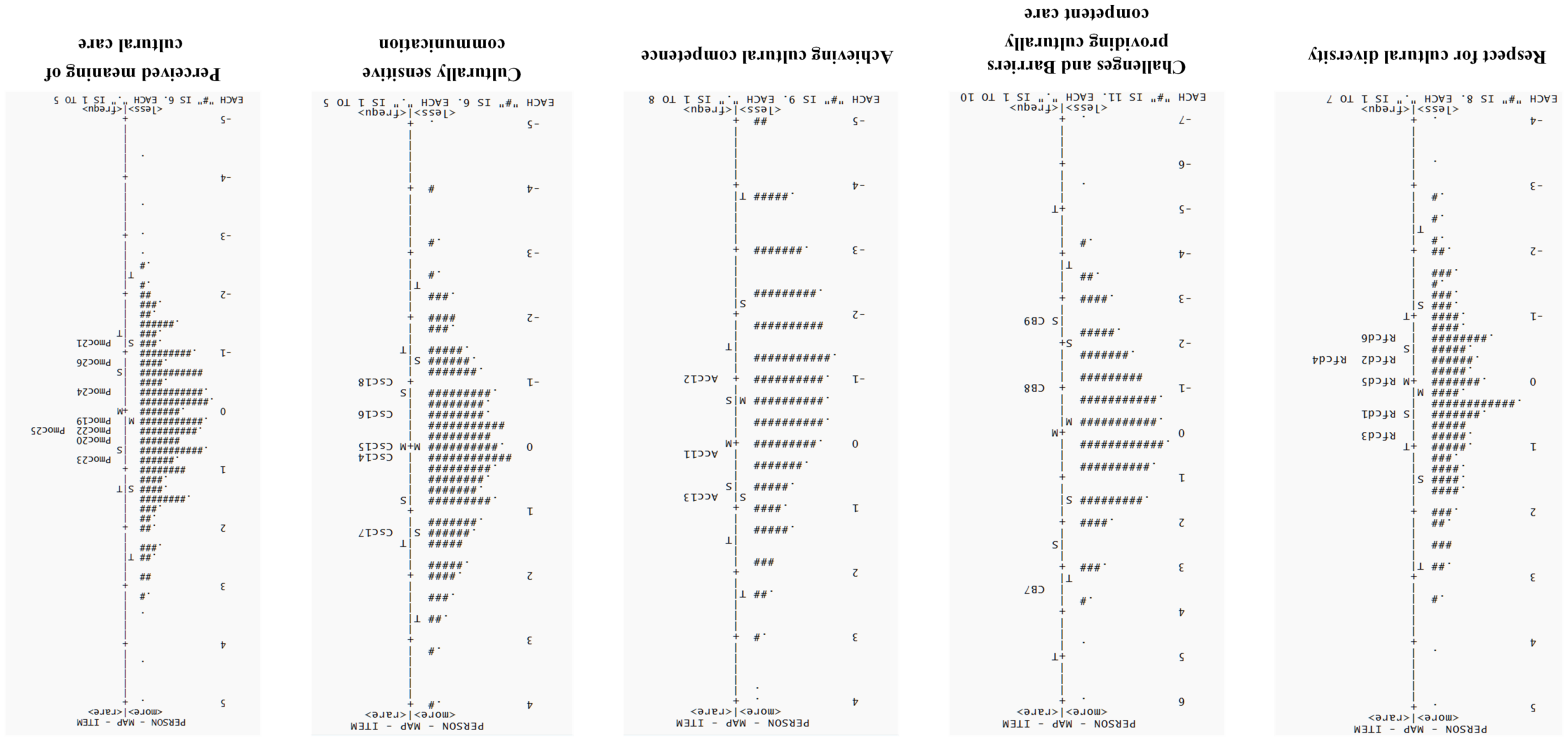
Wright’s person-item map for the C-BENEFITS-CCCSAT.

Wright’s map, which converts the original scores of individual ability and item difficulty into logit values on the same scale, visually illustrates the suitability of items for individuals (40). It serves as one of the key indicators of the overall quality of the scale. The map simultaneously displays the ability levels of the nursing students who took the test and the difficulty of all the items on the C-BENEFITS-CCCSA scale. Ideally, the mean value for both sides should be close to 0, with the difference between them being less than 1 logit (41). A difference greater than 1 logit typically indicates a mismatch between individual ability and item difficulty, suggesting that the individual may not be a good fit for the test. A difference of 1 logit often signifies that the individual’s ability does not align with the difficulty level of the items. The “S” and” T” represent one and two times the standard deviation, respectively. On the left side of the map, the distribution of individual abilities is shown, with higher positions indicating greater ability. On the right side, the distribution of item difficulty is displayed, with higher positions reflecting more difficult items. Figure 2 demonstrates that the overall fit between individual ability and item difficulty across the five dimensions was good, with a similar distribution. The difference in the mean values of the measures did not exceed 1 logit, and the distribution of individual abilities was nearly normal, with most participants concentrated in the middle ability range. The majority of the items on the scale were also located in this region, suggesting that the scale is appropriate for most nursing students. However, the scale’s overall difficulty did not fully accommodate nursing students across all ability ranges. Future studies could consider adding items with higher and lower difficulty levels, as well as adjusting the difficulty intervals between items.

#### 3.3.5 Response category fit

**Figure 3:**
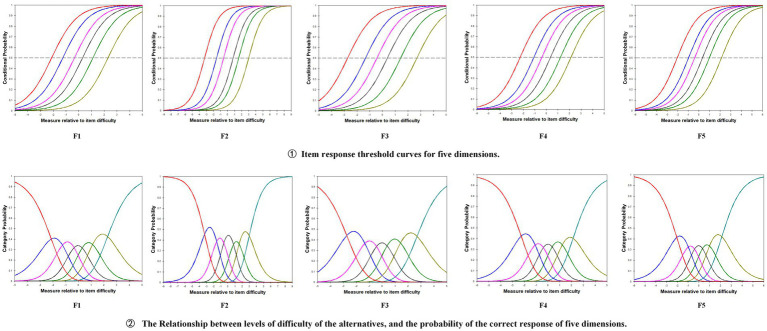
The item characteristic curve of the five dimensions of the C-BENEFITS-CCCSAT (F1: Respect for cultural diversity; F2: Challenges and barriers providing culturally competent care; F3: Achieving cultural competence; F4: Culturally sensitive communication; F5: Perceived meaning of cultural care).

The C-BENEFITS-CCCSAT includes seven response categories: “Strongly Disagree,” “Disagree,” “Somewhat Disagree,” “Neutral,” “Somewhat Agree,” “Agree,” and “Strongly Agree.” Table 6 presents the percentage of occurrences for each response category, the selection difficulty parameters, and the mean square (MNSQ) values for both the infit and outfit statistics. As shown in the table, the difficulty levels of the response categories were calibrated to follow the expected ascending order. According to the infit and outfit statistics, all response categories were statistically appropriate, with the MNSQ values falling within the acceptable range (0.6–1.4) (34). Given that these values represent the ideal fit for infit and outfit statistics, the C-BENEFITS-CCCSAT demonstrated a high degree of fit for the individuals, items, and categories within the rating scale model. Figure 3 (① F1–F5) illustrates the thresholds of the C-BENEFITS-CCCSAT ordered by difficulty, with nearly identical discrimination across the response options. This aligns with the assumptions of the rating scale model, where the difficulty parameter is a key factor influencing the probability of a correct response. Specifically, as difficulty increases, the likelihood of an individual answering a question correctly decreases, while the discrimination parameter remains consistent across all items, indicating that the scale’s ability to differentiate across various difficulty levels is well-balanced (42). In addition, Figure 3 (② F1–F5) demonstrates that the difficulty of the response options increased progressively, with the most difficult option being the correct one. This suggests that the scale effectively reflected the varying ability levels of the respondents. As the ability level increased, the probability of selecting more difficult options also increased, with the most difficult option (typically representing “Strongly Agree” or similar) ultimately becoming the correct answer. This indicates that the scale can effectively differentiate between different trait levels, supporting the assumptions of the scale’s design. These findings demonstrate that the C-BENEFITS-CCCSAT is designed to effectively distinguish between varying levels of ability and item difficulty, making it suitable for a wide range of nursing student populations (43). The peak values of the five-dimensional item characteristic curves were all differentiated from each other and in the same order, indicating that there is a good degree of differentiation among the dimensional items.

**Table 6:** Response category fit for the C-BENEFITS-CCCSAT.

	F1					F2				F3			
Response category	Score	PACO	RCDP	Infit	Outfit	PACO	RCDP	Infit	Outfit	PACO	RCDP	Infit	Outfit
Strongly Disagree	1	7%	−1.88	1.03	1.03	20%	−4.50	1.07	1.06	15%	−2.80	1.08	1.05
Disagree	2	12%	−1.25	0.96	0.97	14%	−2.77	1.05	1.38	21%	−1.85	0.98	0.99
Somewhat Disagree	3	17%	−0.54	1.00	0.97	10%	−1.16	0.90	1.09	20%	−1.01	0.96	0.95
Neutral	4	19%	0.01	0.99	0,97	13%	0.27	0.91	0.90	17%	−0.19	0.94	0.92
Somewhat agree	5	19%	0.65	0.96	0,97	13%	1.36	0.87	0.86	14%	0.61	0.91	0.90
Agree	6	17%	1.35	1.00	0.99	16%	2.29	0.90	1.03	9%	1.42	1.06	1.07
Strongly agree	7	10%	2.17	0.98	0.99	14%	3.58	0.94	0.96	4%	2.39	0.92	0.95

	F4					F5				–			
Response Category	Score	PACO	RCDP	Infit	Outfit	PACO	RCDP	Infit	Outfit	–	–	–	–
Strongly Disagree	1	9%	−2.22	1.03	1.02	8%	−1.81	1.00	1.00	–	–	–	–
Disagree	2	15%	−1.32	0.94	0.97	14%	−1.13	0.97	0.95	–	–	–	–
Somewhat Disagree	3	17%	−0.65	0.97	0.99	16%	−0.58	0.93	0.95	–	–	–	–
Neutral	4	18%	0.05	0.91	0.86	18%	0.02	0.99	1.03	–	–	–	–
Somewhat Agree	5	18%	0.70	0.97	1.00	18%	0.61	0.96	0.96	–	–	–	–
Agree	6	14%	1.35	1.02	1.06	16%	1.22	1.06	1.05	–	–	–	–
Strongly Agree	7	10%	2.14	1.05	1.04	10%	2.08	1.00	1.00	–	–	–	–

PACO, percentage of the appearance of the category in the observed data; RCDP, Response category difficulty parameter; F1, Respect for cultural diversity; F2, Challenges and barriers providing culturally competent care; F3, Achieving cultural competence; F4, Culturally sensitive communication; F5, Perceived meaning of cultural care.

#### 3.3.6 Item function test (DIF)

Differences in item functioning refer to the variation in responses to a given item between individuals of the same ability level across different subgroups (44). When the absolute difference between two groups exceeds 1 logit, it is considered a substantial difference in contrast, indicating that the item is biased (32). The present study found that the DIF contrast values below 0.5 logit indicated measurement invariance in the item functioning test performed among the nursing students from the subgroups with or without cross-culturally relevant training or education (45). These results suggest that the C-BENEFITS-CCCSAT is unbiased in measuring populations with different characteristics (Table 7).

**Table 7:** DIF analysis for the C-BENEFITS-CCCSAT based on whether the participants had attended any courses or training related to cross-cultural care.

WPHACOTCCC	Items	DIF Measure	DIF score	DIF size	DIF SE	DIF t	DIF Contrast	*P*-value
Yes	Rfcd1	0.15	−0.01	0.00	0.03	0.00	0.00	1.000
No	Rfcd1	0.15	0.02	0.00	0.03	0.00	0.00	1.000
Yes	Rfcd2	−0.23	−0.07	0.03	0.03	1.12	0.07	0.261
No	Rfcd2	−0.30	0.08	−0.04	0.03	−1.17	−0.07	0.242
Yes	Rfcd3	0.24	−0.03	0.00	0.03	0.00	0.00	1.000
No	Rfcd3	0.24	0.04	0.00	0.03	0.00	0.00	1.000
Yes	Rfcd4	−0.22	−0.02	0.00	0.03	0.00	0.00	1.000
No	Rfcd4	−0.22	0.02	0.00	0.03	0.00	0.00	1.000
Yes	Rfcd5	−0.08	0.03	0.00	0.03	0.00	0.00	1.000
No	Rfcd5	−0.08	−0.03	0.00	0.03	0.00	0.00	1.000
Yes	Rfcd6	−0.45	0.05	−0.02	0.03	−0.85	−0.06	0.394
No	Rfcd6	−0.39	−0.07	0.03	0.03	1.01	0.06	0.312
Yes	CB7	1.40	0.00	0.00	0.05	0.00	0.00	1.000
No	CB7	1.40	0.01	0.00	0.05	0.00	0.00	1.000
Yes	CB8	−0.25	−0.04	0.00	0.03	0.00	0.02	1.000
No	CB8	−0.27	0.05	−0.02	0.03	0.70	−0.02	0.483
Yes	CB9	−0.71	−0.05	0.03	0.03	0.92	0.06	0.360
No	CB9	−0.77	0.05	−0.03	0.04	−0.87	−0.06	0.382
Yes	Acc11	0.31	−0.03	0.00	0.03	0.00	0.00	1.000
No	Acc11	0.31	0.04	0.00	0.03	0.00	0.00	1.000
Yes	Acc12	−0.06	−0.01	0.00	0.03	0.00	0.00	1.000
No	Acc12	−0.06	0.01	0.00	0.03	0.00	0.00	1.000
Yes	Acc13	0.06	0.06	−0.03	0.03	−1.00	−0.06	0.316
No	Acc13	0.57	−0.06	0.03	0.03	0.96	0.06	0.337
Yes	Csc14	0.63	0.00	0.00	0.03	0.00	0.00	1.000
No	Csc14	0.07	0.00	0.00	0.03	0.00	0.00	1.000
Yes	Csc15	0.07	−0.01	0.00	0.03	0.00	0.00	1.000
No	Csc15	0.01	0.01	0.00	0.03	0.00	0.00	1.000
Yes	Csc16	0.01	−0.04	0.00	0.03	0.00	0.02	1.000
No	Csc16	−0.27	0.05	−0.02	0.03	−0.68	−0.02	0.498
Yes	Csc17	−0.29	−0.08	0.04	0.03	1.40	0.10	0.162
No	Csc17	0.59	0.10	−0.05	0.03	1.63	−0.10	0.104
Yes	Csc18	0.50	−0.03	0.00	0.05	0.00	−0.06	1.000
No	Csc18	−0.46	0.03	0.00	0.05	0.00	0.06	1.000
Yes	Pmoc19	−0.46	0.06	−0.03	0.03	−0.99	0.00	0.323
No	Pmoc19	0.01	−0.07	0.03	0.03	1.06	0.00	0.290
Yes	Pmoc20	0.07	0.03	0.00	0.03	0.00	0.00	1.000
No	Pmoc20	0.18	−0.03	0.00	0.04	0.00	0.00	1.000
Yes	Pmoc21	0.18	0.02	0.00	0.03	0.00	−0.05	1.000
No	Pmoc21	−0.59	−0.02	0.00	0.03	0.00	0.05	1.000
Yes	Pmoc22	−0.59	0.06	−0.03	0.03	−0.94	0.00	0.346
No	Pmoc22	0.07	−0.07	0.03	0.03	0.99	0.00	0.321
Yes	Pmoc23	0.13	0.02	0.00	0.03	0.00	0.00	1.000
No	Pmoc23	0.30	−0.02	0.00	0.03	0.00	0.00	1.000
Yes	Pmoc24	0.30	0.02	0.00	0.03	0.00	0.00	1.000
No	Pmoc24	−0.23	−0.02	0.00	0.03	0.00	0.00	1.000
Yes	Pmoc25	−0.23	0.02	0.00	0.03	0.00	0.00	1.000
No	Pmoc25	0.11	−0.02	0.00	0.03	0.00	0.00	1.000
Yes	Pmoc26	−0.49	0.06	−0.03	0.03	−1.01	−0.07	0.313
No	Pmoc26	−0.43	−0.08	0.04	0.03	1.19	0.07	0.234

WPHACOTCCC, whether participants had attended any courses or training related to cross-cultural care; DIF Score is the difference between the observed and the expected average observations. DIF Measure is the difficulty of the item for the category, with all else held constant. DIF Size is the difference between the DIF measure for a certain category and the baseline difficulty. DIF SE is the standard error of the second DIF measure. DIF t is the DIF contrast divided by the SE of the two DIF measures. It is equivalent to the Mantel–Haenszel significance test but has the advantage of allowing missing data.

## 4 Discussion

Cross-cultural care aims to address the unique needs of patients from diverse cultural backgrounds by providing culturally competent care (46). However, the implementation of intercultural care in healthcare settings faces numerous challenges (47), one of which is the lack of effective assessment tools. This study translated advanced foreign tools for assessing transcultural nursing skills, cultural competence, and sensitivity, with the goal of providing a scientifically valid and effective assessment instrument for the healthcare field. By doing so, it aimed to promote the development of transcultural nursing, enhance the quality of care, and better meet the diverse nursing needs of patients. However, we encountered several challenges during the study. Differences in expression between languages may have caused some items on the scale to fail in accurately conveying the original measurement intent after translation, potentially affecting their validity and reliability across cultural contexts. To address this, we employed a “direct translation-back translation” method and conducted two rounds of expert review to ensure the scale’s applicability and cultural sensitivity across various languages and cultures (48). Despite these efforts, during the first validation, we found that item 10, “I have concerns about culturally competent care,” had a low factor loading (0.44) (49) and poor fit indices (infit MNSQ: 3.33, outfit MNSQ: 3.54) (50). This may be due to differences in the understanding of the term “concerns” between Chinese and Western cultures, which suggests that the item might not effectively convey its intended meaning within Chinese nursing culture. In addition, the item may have been conceptually ambiguous, leading to misinterpretation by the respondents, which affected the scale’s overall performance. After consulting with the experts, the research team decided to remove this item. Following its deletion, the C-BENEFITS-CCCSAT was re-evaluated using the CTT and Rasch models.

The CTT analysis demonstrated that the C-BENEFITS-CCCSAT exhibited strong reliability and validity overall. Cronbach’s *α* was 0.80, with the dimension reliability values ranging from 0.700 to 0.905 and a split-half reliability value of 0.837. The test–retest reliability value was 0.881, and the S-CVI value was 0.928, with the I-CVI values ranging from 0.83 to 1.00. The CFA revealed a good model fit, with a CMIN/DF ratio of 1.071, a RMSEA value of 0.08, and the CFI, NFI, IFI, and TLI all exceeding 0.9. The factor loadings for all items were ≥ 0.50. The CR values ranged from 0.700 to 0.88, and the AVE values ranged from 0.420 to 0.55, meeting the criteria for convergent and discriminant validity. It is worth noting that the validity of the calibration correlations between the C-BENEFITS-CCCSAT and the C-SFCCSN was moderate. This may be attributed to differences in the dimensionality and theoretical frameworks of the two scales, despite both assessing intercultural caregiving competence. These differences likely contributed to the weaker correlations between certain dimensions. In addition, variations in the working environments of the sample may have influenced the correlation, thus limiting the comprehensiveness of the validity test. The Rasch analysis showed that the C-BENEFITS-CCCSAT had an item reliability of 1.00 and a person reliability of 0.76–0.89, both exceeding the 0.7 threshold, indicating strong reliability. The item separation index values ranged from 17.37 to 60.34, and the person separation index values ranged from 1.76 to 2.89, both exceeding the minimum criterion of 1.5, suggesting a good representation of both the items and persons. The infit MNSQ values for the items ranged from 0.87 to 1.08, and the outfit MNSQ values ranged from 0.86 to 1.27, meeting the model fit requirements. The PT-Measure Corr values ranged from 0.68 to 0.84, exceeding the minimum reference value of 0.5, showing that the items closely aligned with the measurement objectives and that the data fit the model well. The fit between individual ability and item difficulty was good, with the distribution of ability approximating a normal curve. The majority of the participants were clustered in the intermediate ability range, where most items were also located. The difficulty of the response categories followed the expected order, with neither the infit nor outfit statistics exceeding the acceptable fit range (0.6–1.4), indicating a good fit across the individuals, items, and categories. Furthermore, the DIF contrast value was below 0.5 logit in the subgroup with cross-cultural training experience, indicating no measurement bias across the groups. The combination of CTT and IRT not only validates the psychometric properties of the scale but also emphasizes the importance of assessing cross-cultural nursing competence in both nursing education and clinical practice.

The development of cross-cultural nursing competence is essential in nursing education (51). However, many nursing students still face gaps in cultural sensitivity and adaptation, which can impact their future clinical practice in multicultural settings. The C-BENEFITS-CCCSAT serves as a standardized tool to help educators identify and address these gaps (7). Specifically, the scale can be used to assess students’ cultural competence at different stages of the nursing curriculum. For example, at the start of the course, educators can use the scale to establish a baseline assessment of students’ intercultural competence. If the results show low scores in dimensions such as “respect for cultural diversity,” it indicates the need for additional instruction and training. Educators can then develop individualized teaching plans, such as case studies, role-playing, or scenarios (52). At the end of the course, the scale can be used again to assess student progress, allowing for data-driven adjustments to future course designs. This approach enhances the accuracy and effectiveness of nursing education, promoting the development of culturally sensitive nursing professionals.

In addition to identifying gaps in students’ cultural competence, the scale can also serve as an assessment tool in nursing courses, particularly for formative and summative assessments. Educators can use the scale to evaluate students’ cultural competence midway through a nursing program focused on intercultural nursing. If results show low scores in areas such as ‘understanding the impact of cultural context on health beliefs,’ educators can adjust the curriculum to include discussions on culturally relevant health beliefs or invite diverse cultural experts to share clinical experiences.

At the end of the program, educators can use the C-BENEFITS-CCCSAT to assess students’ final performance in cultural competence. During graduation assessments, students can self-assess their cultural competence using the scale, while faculty members can evaluate students and incorporate the results into the overall teaching quality assessment. This ensures that nursing education focuses not only on theoretical knowledge but also on meeting the expected standards for cultural sensitivity and intercultural nursing practice (53). In addition, the scale can be integrated into clinical placement assessments. Clinical supervisors can use it to evaluate students’ performance in communicating with culturally diverse patients and developing care plans during their placements. This allows for monitoring students’ intercultural competence in real-world nursing environments and provides targeted feedback to help them improve their cultural nursing skills.

Finally, the development of cultural competence is crucial to ensure that future nurses can provide high-quality care in multicultural settings. Culturally sensitive nursing involves not only effective verbal communication but also a deep respect for patients’ cultural beliefs, lifestyles, and health perspectives (54). Nurses must recognize cultural differences, understand patients’ needs, and adapt their care accordingly (55). For example, in clinical practice, nurses may encounter language barriers, such as when a diabetic patient from a non-native-speaking country lacks knowledge about diabetes management and struggles to understand hospital health education materials. A culturally competent nurse would recognize the challenges posed by the language barrier and use translation services or more accessible methods to help the patient understand their condition (56). In addition, nurses may use visual teaching tools tailored to the patient’s cultural background, ensuring the patient actively engages in managing their diabetes. By incorporating the scale into clinical placements, educators can assess students’ communication skills and cultural sensitivity with patients from diverse cultural backgrounds, providing timely feedback. This not only enhances students’ intercultural nursing competence but also offers an effective tool for assessing cultural competence in future nursing practice.

## 5 Conclusion

This study successfully validated the C-BENEFITS-CCCSAT using a rigorous methodology that integrated CTT and IRT. The CTT analysis demonstrated strong reliability and validity, while the Rasch analysis confirmed the scale’s good fit and measurement precision. By combining these two approaches, we ensured the scale’s robustness in assessing cultural competence, sensitivity, and skills. The C-BENEFITS-CCCSAT is a valuable tool for nursing educators to identify and address gaps in students’ cross-cultural competence. It should be noted that despite the deletion of item 10, the five-factor structure of the Chinese version of the scale is consistent with the original scale, which is perfect. It can be applied in both educational settings to monitor progress and in clinical settings to evaluate students’ ability to deliver culturally competent care. This approach provides a reliable framework for enhancing nursing education and improving care in multicultural environments. Future studies should test the scale in diverse populations to further establish its applicability and generalizability.

## 6 Limitations

The sample in this study was limited to Chinese nursing students, which might have affected the generalizability of the findings to other cultural or geographic contexts. Due to differences in the cultural and social backgrounds, the nursing students from different regions or countries might have had significant variations in their understanding and practice of cultural competence. Therefore, the applicability of the results may be somewhat limited. Future research could validate the C-BENEFITS-CCCSAT in different cultural and geographic contexts to assess its applicability and reliability globally, thus improving its generalizability. Furthermore, while this study provides a valuable cultural competence assessment tool for nursing education in China, given the diversity in global nursing education, the scale may require some adjustments to suit the cultural characteristics of other countries and regions. For example, some items may need to be localized to better align with the realities of different cultural groups. Therefore, future research should consider expanding the C-BENEFITS-CCCSAT to nursing students from diverse cultural backgrounds and explore the challenges and necessary modifications when using this tool in international nursing education. In conclusion, while this study provides a useful tool for assessing cross-cultural nursing competence, its limitations should be thoroughly discussed and addressed in future international research to ensure that the tool can be widely applied in nursing education and clinical practice across different cultural and geographic settings.

## Data Availability

The raw data supporting the conclusions of this article will be made available by the authors, without undue reservation.

## Footnotes

^1^ https://www.wjx.cn/
